# Spin-Hall conductivity and Hall angle in a two-dimensional system with impurities in the presence of spin–orbit interactions

**DOI:** 10.1038/s41598-022-18042-w

**Published:** 2022-08-20

**Authors:** Hemant Kumar Sharma, Shreekantha Sil, Ashok Chatterjee

**Affiliations:** 1 School of Physics, University of Hyderabad, Hyderabad, Telangana India; 2 Department of Physics, Visva Bharati University, Bolpur, West Bengal India

**Keywords:** Nanoscience and technology, Physics

## Abstract

We investigate the spin-torque-dependent Spin Hall phenomenon in a two-dimensional tight-binding system in the presence of Rashba and Dresselhaus spin–orbit interactions and random static impurities. We employ the Matsubara Green function techniques to calculate the relaxation time caused by the scattering of electrons by impurities. The longitudinal and transverse conductivities are next calculated with the help of the Kubo formalism. We have also calculated the spin Hall angle for the present model and studied its dependence on spin–orbit interactions and impurity strength. Finally, we explore the effect of interplay between the Rashba and Dresselhaus interactions on the spin-Hall effect.

## Introduction

With recent development in the field of spin-based devices, the subject of Spintronics has become an active area of research in low-dimensional physics for its potential applications in spin filters, field-effect spin transistors, information processing, and mass storage^1,2^, magnetic recording, sensors and so on. Spin transport plays a key role in spintronic devices and causes a spin current in a similar manner the charge transport gives the charge current. Methods like injecting ferromagnetic atoms in a non-ferromagnetic material^3–10^ have been used widely in the past to get spin current. However, this method is efficacious only when the magnetic field is strong. An alternative approach has been proposed by Sharma et al.^11^ to produce spin current in the GaAs/AlGaAs hetero-structure. Here the basic idea is that in the GaAs/AlGaAs hetero-structure cavity, the spin–orbit interaction couples the electron’s spin and orbital motion making the electrons of the system spin-polarized^11^. The study of intrinsic Spin-Hall effect in p-doped semiconductors by Murakami et al.^12^ and in a two-dimensional (2D) electron gas by Sinova et al.^13^ with a substantial Rashba spin–orbit coupling has brought the subject of spintronics to the forefront of current research. The intrinsic spin-Hall effect refers to a non-dissipative spin current flowing normal to the driving electric field when the spin–orbit interaction (SOI) dominates over the quantum collisions caused by the disorder. This is in contrast to the extrinsic Spin-Hall effect proposed by Hirsch^14^ and Zhang^15^, where the spin current is generated by the spin–orbit dependent scattering from the impurity. Also, the effect of random disorders on the spin-Hall effect has been widely studied over time. The impurities considered have mainly been magnetic^16–19^, and also static random impurities^20–29^. Inoue et al.^30,31^ have shown that in a two-dimensional electron gas, spin accumulation can be achieved by applying a bias and have calculated the diffusive conductance tensor. They have also demonstrated that vertex correction in SHC causes SHC to vanish.

In most of the earlier works on spin transport mentioned above, the spin current has been defined in the conventional way and is given by $\frac{\hbar }{4}\left\{ {{\varvec{v}},{\varvec{\sigma}}} \right\}$^13,31^ In the presence of SOIs, this definition suffers from two fundamental drawbacks. First, the conservation of spin magnetic moment no longer holds and secondly, the spin current is found to be finite even in a localized state. To avoid these difficulties, Shi et al.^32,33^ have put forward an alternative definition for the current. They define the current as a time derivative of the polarization operator, which differs from the conventional definition by the torque dipole term. Here the torque dipole term in the case of spin current is given by the expectation value of the rate of change of spin and arises when the spin magnetic moment of the system is not conserved. Recently, we have studied the effect of disorder on the longitudinal charge and spin currents in a 2D system described by the tight-binding model (TBM) incorporating the Rashba SOI (RSOI) effect^34^. Later we have also studied the same model taking into account the effects of both RSOI and Dresselhaus SOI (DSOI)^35^. DSOI arises because of the breaking of bulk inversion symmetry and its strength (which is not tunable) is of the same order as RSOI’s and is usually present in almost all systems. Therefore, the incorporation of DSOI is important for the proper understanding of any SOI effect.

In this paper, we purport to examine the role of RSOI, DSOI, and the static random disorder on the longitudinal spin conductivity (LSC) and the transverse spin conductivity in a 2D TBM system using Shi’s approach. The transverse spin conductivity is usually referred to as the spin Hall conductivity (SHC). For the sake of completeness, we shall also calculate the corresponding charge conductivities. We finally calculate the ratio: SHC/LCC which is known as the spin-Hall angle^36^ and study its dependence on various parameters.

## Model and formalism

We consider a 2D TBM for a system of electrons with RSOI and DSOI^34,37^ in the presence of static impurities which will be considered random. The total Hamiltonian is given by

1 $$ H = H_{0} + H_{imp} , $$

with

2 $$ \begin{aligned} H_{0} & = \varepsilon_{0} \mathop \sum \limits_{i} c_{i}^{\dag } c_{i} + t \mathop \sum \limits_{{\left\langle {i,j} \right\rangle }} \left[ {c_{i}^{\dag } c_{j} + h.c.} \right] - i\alpha_{R} \mathop \sum \limits_{{\left\langle {i,j} \right\rangle }} \left[ {c_{ix,iy}^{\dag } \sigma^{y} c_{ix + 1,iy} + h.c.} \right] + i\alpha_{R} \mathop \sum \limits_{{\left\langle {i,j} \right\rangle }} \left[ {c_{ix,iy}^{\dag } \sigma^{x} c_{ix + 1,iy} + h.c.} \right] \\ & \quad + i\beta_{D} \mathop \sum \limits_{{\left\langle {i,j} \right\rangle }} \left[ {c_{ix,iy}^{\dag } \sigma^{x} c_{ix + 1,iy} + h.c.} \right] - \beta_{D} \mathop \sum \limits_{{\left\langle {i,j} \right\rangle }} \left[ {c_{ix,iy}^{\dag } \sigma^{y} c_{ix,iy + 1} + h.c.} \right] , \\ \end{aligned} $$

3 $$ H_{imp} = \mathop \sum \limits_{i} \epsilon_{i} c_{i}^{\dag } c_{i} = \mathop \sum \limits_{i} v \delta \left( {R_{i} - r_{l} } \right)c_{i}^{\dag } c_{i} . $$

where $$c_{i}^{\dag } = \left( {\begin{array}{*{20}c} {c_{i \uparrow }^{\dag } } & {c_{i \downarrow }^{\dag } } \\ \end{array} } \right)$$, and $$c_{i} = \left( {\begin{array}{*{20}c} {c_{i \uparrow } } \\ {c_{i \downarrow } } \\ \end{array} } \right)$$ denote the creation and annihilation operators for the spin up and spin down electrons, $\varepsilon_{0}$ and* t* refer respectively to the onsite energy and the hopping integral, $\left\langle {i,j} \right\rangle$ runs over all the nearest neighbors sites, $\sigma^{x} ,\sigma^{y} ,\sigma^{z}$ are the Pauli matrices, $\alpha_{R}$ and $\beta_{D}$ denote the RSOI and DSOI strengths respectively, $R_{i}$ and $r_{l}$ refer to the electron and impurity positions respectively and the electron–impurity strength is measured by $v$. When $H_{imp} = 0$, the Hamiltonian *H* can be diagonalized by a unitary transformation and spin degeneracy is lifted by the spin–orbit coupling leading to two non-degenerate states corresponding to two types of electrons. To show this, we perform the transformation:

4 $$ U\left( {\varvec{k}} \right)\left( {\begin{array}{*{20}c} {c_{{{\varvec{k}} \uparrow }} } \\ {c_{{{\varvec{k}} \downarrow }} } \\ \end{array} } \right) = \left( {\begin{array}{*{20}c} {\alpha_{{1,{\varvec{k}}}} } \\ {a_{{2,{\varvec{k}}}} } \\ \end{array} } \right) = \alpha_{{\varvec{k}}} , $$

with

4a $$ U\left( {\varvec{k}} \right) = \left( {\begin{array}{*{20}c} 1 & {p_{{\varvec{k}}} } \\ { - p_{{\varvec{k}}}^{\user2{*}} } & 1 \\ \end{array} } \right),\quad p_{{\varvec{k}}} = \zeta \left( {\varvec{k}} \right)/\left| \zeta \right|, $$

and write the Hamiltonian in terms of the new operators as:

5 $$ H_{0} = \mathop \sum \limits_{{\varvec{k}}} \left[ {\epsilon_{{1,{\varvec{k}}}} \alpha_{{1,{\varvec{k}}}}^{\dag } \alpha_{{1,{\varvec{k}}}} + \epsilon_{{2,{\varvec{k}}}} \alpha_{{2,{\varvec{k}}}}^{\dag } \alpha_{{2,{\varvec{k}}}} } \right] $$

Energetics of the two types of electrons are given by

6 $$ \epsilon_{{1,2,{\varvec{k}}}} = \epsilon_{{\varvec{k}}} \pm 2 \left| {\zeta \left( {\varvec{k}} \right)} \right|, $$

with

6a $$ \zeta \left( {\varvec{k}} \right) = \left( {\alpha_{R} sink_{y} + \beta_{D} sink_{x} } \right) + i\left( {\alpha_{R} sink_{x} + \beta_{D} sink_{y} } \right),\quad \epsilon_{{\varvec{k}}} = \epsilon_{0} + 2t\left( {cos{\text{k}}_{{\text{x}}} + cos{\text{k}}_{{\text{y}}} } \right). $$

## Relaxation time

The effect of impurity is studied by calculating the relaxation time involved in the scattering of electrons by the impurities. To simplify, we first write the impurity Hamiltonian in terms of the transformed operators $\alpha_{{1,{\varvec{k}}}}$ and $a_{{2,{\varvec{k}}}}$ as

7 $$ H_{imp} = \mathop \sum \limits_{{{\varvec{kk}}^{{\prime }} }} \left[ {V_{{\user2{kk^{\prime}}}}^{11} \alpha_{{1,{\varvec{k}}}}^{\dag } \alpha_{{1,\user2{k^{\prime}}}} + V_{{\user2{kk^{\prime}}}}^{12} \alpha_{{1,{\varvec{k}}}}^{\dag } \alpha_{{2,\user2{k^{\prime}}}} + V_{{\user2{kk^{\prime}}}}^{21} \alpha_{{2,{\varvec{k}}}}^{\dag } \alpha_{{1,\user2{k^{\prime}}}} + V_{{\user2{kk^{\prime}}}}^{22} \alpha_{{2,{\varvec{k}}}}^{\dag } \alpha_{{2,\user2{k^{\prime}}}} } \right], $$

where

7a $$ V_{{{\varvec{kk}}^{{\prime }} }}^{11} = \frac{1}{2N}\mathop \sum \limits_{l} ve^{{i\left( {{\varvec{k}} - {\varvec{k}}^{{\prime }} } \right).r_{l} }} \left( {1 + p_{{\varvec{k}}} p_{{{\varvec{k}}^{{\prime }} }}^{*} } \right),\quad V_{{{\varvec{kk}}^{{\prime }} }}^{12} = \frac{1}{2N}\mathop \sum \limits_{l} ve^{{i\left( {{\varvec{k}} - {\varvec{k}}^{{\prime }} } \right).r_{l} }} \left( {p_{{\varvec{k}}} - p_{{{\varvec{k}}^{{\prime }} }} } \right), $$

7b $$ V_{{{\varvec{kk}}^{{\prime }} }}^{21} = \frac{1}{2N}\mathop \sum \limits_{l} ve^{{i\left( {{\varvec{k}} - {\varvec{k}}^{{\prime }} } \right).r_{l} }} \left( {p_{{k^{{\prime }} }}^{*} - p_{k}^{*} } \right),\quad V_{{{\varvec{kk}}^{{\prime }} }}^{22} = \frac{1}{2N}\left( {p_{{k^{{\prime }} }}^{*} - p_{k}^{*} } \right)\left( {1 + p_{{{\varvec{k}}^{{\prime }} }} p_{{\varvec{k}}}^{*} } \right). $$

In the presence of impurities, the charge carrier will acquire a relaxation time. The relaxation time can be calculated from the imaginary part of the self-energy. The relevant Green function for the electron-impurity system is given by

8 $$ G\left( {k,\tau } \right) = \mathop \sum \limits_{l = 1}^{\infty } \left( { - 1} \right)^{l} \mathop \int \limits_{0}^{\beta } d\tau_{1} \ldots \ldots \ldots \mathop \int \limits_{0}^{\beta } d\tau_{n} *Tr\left[ {\alpha_{1,k}^{\dag } \left( \tau \right)V\left( {\tau_{1} } \right)V\left( {\tau_{2} } \right) \ldots \ldots \ldots V\left( {\tau_{l} } \right)\alpha_{1,k} \left( 0 \right)} \right] $$

Here we will calculate Green function to the second order, as the first order i.e. *l* = 1, give a constant shift in energy. Second order Green function for our Hamiltonian is given by

9 $$ \begin{aligned} G\left( {k,\tau } \right) & = \sum\limits_{{k,k^{\prime}}} {\int\limits_{0}^{\beta } {\int\limits_{0}^{\beta } {d\tau_{1} d\tau_{2} \left\langle {T_{\tau } \left[ {\alpha_{1,k} \left( {V_{{k_{1} k_{2} }}^{11} \alpha_{{1,k_{1} }}^{\dag } \left( {\tau_{1} } \right)\alpha_{{1,k_{2} }} \left( {\tau_{1} } \right) + V_{{k_{1} k_{2} }}^{12} \alpha_{{1,k_{1} }}^{\dag } \left( {\tau_{1} } \right)\alpha_{{2,k_{2} }} \left( {\tau_{1} } \right)} \right.} \right.} \right.} } } \\ & \quad \left. { + V_{{k_{1} k_{2} }}^{21} \alpha_{{2,k_{1} }}^{\dag } \left( {\tau_{1} } \right)\alpha_{{1,k_{2} }} \left( {\tau_{1} } \right) + V_{{k_{1} k_{2} }}^{22} \alpha_{{2,k_{1} }}^{\dag } \left( {\tau_{1} } \right)\alpha_{{2,k_{2} }} \left( {\tau_{1} } \right)} \right) \\ & \quad \left( {V_{{k^{\prime}_{1} k^{\prime}_{2} }}^{11} \alpha_{{1,k^{\prime}_{1} }}^{\dag } \left( {\tau_{2} } \right)\alpha_{{1,k^{\prime}}} \left( {\tau_{2} } \right) + V_{{k^{2} k^{\prime}}}^{12} \alpha_{{1,k^{2} }}^{\dag } \left( {\tau_{2} } \right)\alpha_{{2,k^{\prime}}} \left( {\tau_{2} } \right)} \right. \\ & \quad \left. {\left. {\left. { + V_{{k^{2} k^{\prime}}}^{21} \alpha_{{2,k^{2} }}^{\dag } \left( {\tau_{2} } \right)\alpha_{{1,k^{\prime}}} \left( {\tau_{2} } \right) + V_{{k^{2} k^{\prime}}}^{22} \alpha_{{2,k^{2} }}^{\dag } \left( {\tau_{2} } \right)\alpha_{{2,k^{\prime}}} \left( {\tau_{2} } \right)} \right)\alpha_{1,k}^{\dag } \left( 0 \right)} \right]} \right\rangle \\ \end{aligned} $$

Using Wicks theorem, the Green function Eq. (9) reduces to

10 $$ G\left( {k,ip_{n} } \right) = \mathop \sum \limits_{{k_{1} }} V_{{kk^{1} }}^{11} V_{{k^{1} p}}^{11} g^{01} \left( {k,ip_{n} } \right)g^{01} \left( {k_{1} ,ip_{n} } \right)g^{01} \left( {k,ip_{n} } \right) + V_{{kk^{1} }}^{12} V_{{k^{1} k}}^{21} g^{01} \left( {k,ip_{n} } \right)g^{02} \left( {k_{1} ,ip_{n} } \right)g^{01} \left( {k,ip_{n} } \right). $$

As the impurities are randomly distributed, we perform impurity averaging over all possible configurations. Considering the distributions of impurities to be uncorrelated in space, we write it as the product of the individual impurity distributions. Since the factor which depends on position is $V_{{kk_{1} }} V_{{k_{1} k}}$ and we can write

11 $$ \overline{{V_{{kk_{1} }} V_{{k_{1} k}} }} = \frac{{v^{2} }}{{4N^{2} }}\left[ {\left[ {\left( {\aleph^{2} - \aleph } \right)\delta_{{kk_{1} }} \delta_{{k_{1} k}} + \aleph } \right] \left[ {\left( {1 + p_{k} p_{{k_{1} }}^{*} } \right)} \right]\left[ {\left( {1 + p_{{k_{1} }} p_{k}^{*} } \right)} \right]} \right] $$

Substituting Eq. (11) in (10) the Green functions reduces to

12 $$ \begin{aligned} G\left( {k,ip_{n} } \right) & = g^{01} \left( {k,ip_{n} } \right)\mathop \sum \limits_{{k_{1} }} \frac{{v^{2} }}{{4{\text{N}}^{2} }}\left[ {\left( {\aleph^{2} } \right)\delta_{{kk_{1} }} \delta_{{k_{1} k}} + \aleph } \right] \times \left[ {\left( {1 + p_{k} p_{{k_{1} }}^{*} } \right)} \right]\left[ {\left( {1 + p_{{k_{1} }} p_{k}^{*} } \right)} \right] \\ & \quad \times g^{01} \left( {k_{1} ,ip_{n} } \right)g^{01} \left( {k,ip_{n} } \right) + g^{01} \left( {k,ip_{n} } \right) \times \mathop \sum \limits_{{k_{1} }} \frac{{v^{2} }}{{4{\text{N}}^{2} }}\left[ {\left( {\aleph^{2} } \right)\delta_{{kk_{1} }} \delta_{{k_{1} k}} + \aleph } \right] \\ & \quad \times \left[ {\left( {p_{k} - p_{{k_{1} }} } \right)} \right]\left[ {\left( {p_{k}^{*} - p_{{k_{1} }}^{*} } \right)} \right]g^{02} \left( {k_{1} ,ip_{n} } \right)g^{01} \left( {k,ip_{n} } \right) \\ \end{aligned} $$

**Figure 1:**
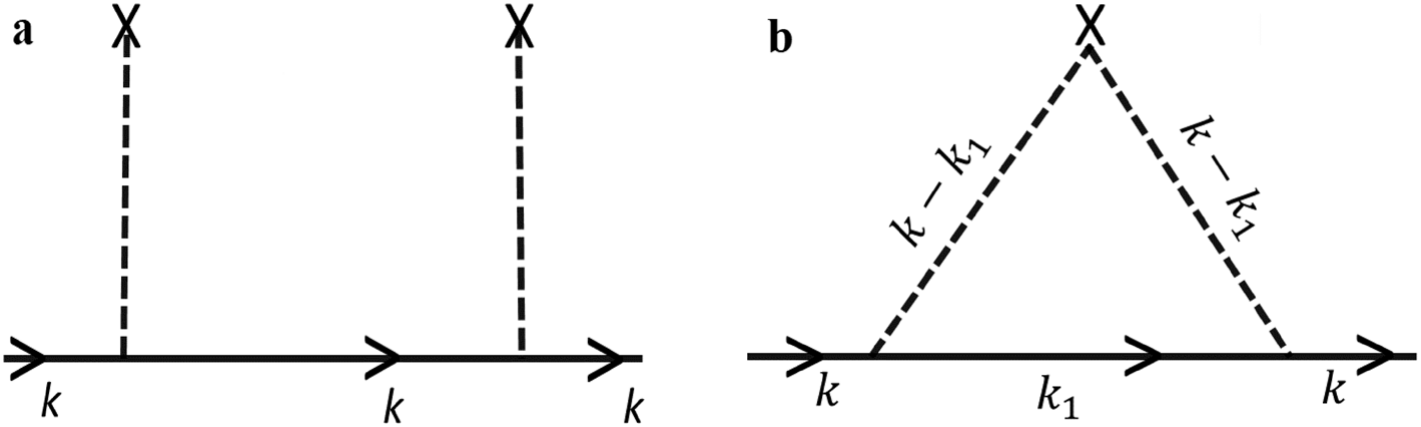
Feynman diagrams for $G\left( {k,ip_{n} } \right)$ for orders *n* = 2.

In Eq. (12) we have contributions to the Green functions from two terms, one from $\aleph$ and another from $\aleph^{2}$ as shown in Fig. 1 where $\aleph$ is the number of impurities present in the system. The contribution from $\aleph^{2}$ (Fig. 1a) will be cancelled as it represents a reducible diagram. The contribution from Fig. 1b is given as

13 $$ \begin{aligned} G\left( {k,ip_{n} } \right) & = g^{01} \left( {k,ip_{n} } \right)\mathop \sum \limits_{{k_{1} }} \frac{{v^{2} }}{{4{\text{N}}}}n\left( {1 + p_{k} p_{{k_{1} }}^{*} } \right)\left( {1 + p_{{k_{1} }} p_{k}^{*} } \right) g^{01} \left( {k_{1} ,ip_{n} } \right) \times g^{01} \left( {k,ip_{n} } \right) \\ & \quad + g^{01} \left( {k,ip_{n} } \right)\mathop \sum \limits_{{k_{1} }} \frac{{v^{2} }}{{4{\text{N}}}}n\left( {p_{k} - p_{{k_{1} }} } \right) \times \left( {p_{k}^{*} - p_{{k_{1} }}^{*} } \right) \times g^{02} \left( {k_{1} ,ip_{n} } \right)g^{01} \left( {k,ip_{n} } \right). \\ \end{aligned} $$

With further simplification, the Green function equation can be rewritten as

14 $$ G\left( {k,ip_{n} } \right) = g^{01} \left( {k,ip_{n} } \right)\Sigma \left( {ip_{n} } \right)g^{01} \left( {k,ip_{n} } \right) $$

where $\Sigma \left( {ip_{n} } \right)$ is the self-energy and is given by

15 $$ \begin{aligned} \Sigma \left( {ip_{n} } \right) & = \frac{{v^{2} }}{4N}\mathop \sum \limits_{{k_{1} }} \left[ {n\left[ {\left( {1 + p_{k} p_{{k_{1} }}^{*} } \right)} \right]\left[ {\left( {1 + p_{{k_{1} }} p_{k}^{*} } \right)} \right]} \right]g^{01} \left( {k_{1} ,ip_{n} } \right) \\ & \quad + \frac{{v^{2} }}{4N}\mathop \sum \limits_{{k_{1} }} \left[ {n\left[ {\left( {p_{k} - p_{{k_{1} }} } \right)} \right]\left[ {\left( {p_{k}^{*} - p_{{k_{1} }}^{*} } \right)} \right]} \right]g^{02} \left( {k_{1} ,ip_{n} } \right). \\ \end{aligned} $$

In Eq. (15), $g^{01} \left( {k_{1} ,ip_{n} } \right)$ and $g^{02} \left( {k_{1} ,ip_{n} } \right)$ denote the Green functions for an electron in the absence of the impurities and having a particular spin

16 $$ g^{01} \left( {k_{1} ,ip_{n} } \right) = \mathop \sum \limits_{{k_{1} }} \frac{1}{{ip_{n} - \epsilon_{{1,k_{1} }} }},\quad g^{02} \left( {k_{1} ,ip_{n} } \right) = \mathop \sum \limits_{{k_{1} }} \frac{1}{{ip_{n} - \epsilon_{{2,k_{1} }} }}. $$

The next step is to perform the analytical continuation: $ip_{n} \to \epsilon + i\eta$. The self-energy is thus given by

17 $$ \Sigma \left( \epsilon \right) = \frac{{v^{2} }}{4N}\mathop \sum \limits_{{k_{1} }} \frac{{\left[ {n\left[ {\left( {1 + p_{k} p_{{k_{1} }}^{*} } \right)} \right]\left[ {\left( {1 + p_{{k_{1} }} p_{k}^{*} } \right)} \right]} \right]}}{{\epsilon - \epsilon_{{1,k_{1} }} + isgn\left( {p_{n} } \right)\eta }} + \frac{{v^{2} }}{4N}\mathop \sum \limits_{{k_{1} }} \frac{{\left[ {n\left[ {\left( {p_{k} - p_{{k_{1} }} } \right)} \right]\left[ {\left( {p_{k}^{*} - p_{{k_{1} }}^{*} } \right)} \right]} \right]}}{{\epsilon - \epsilon_{{2,k_{1} }} + isgn\left( {p_{n} } \right)\eta }}, $$

and the imaginary part of the self-energy gives relaxation time.

18 $$ \frac{1}{{\tau_{k}^{1,2} }} = \frac{2\pi }{\hbar }\frac{{v^{2} n}}{4N} \mathop \sum \limits_{{k_{1} }} \left( {\left[ {\left( {1 + \left| {p_{k} } \right|^{2} \left| {p_{k} } \right|^{2} } \right)} \right]\delta \left( {\epsilon - \epsilon_{{1,2,k_{1} }} } \right) + \left[ {\left| {p_{k} } \right|^{2} + \left| {p_{k} } \right|^{2} } \right]\delta \left( {\epsilon - \epsilon_{{2,1,k_{1} }} } \right)} \right) $$

In the dilute-impurity regime, the average-configuration relaxation times for the two types of electrons may be assumed to be equal. Defining a characteristic time scale $\tau_{0}$ given by: $\tau_{0} = \hbar /t$ and introducing:$y_{i}^{{\prime }} = y_{i} /t,\alpha_{R}^{{\prime }} = \alpha_{R}^{{\prime }} = \alpha_{R} /t,\beta_{D}^{{\prime }} = \beta_{D} /t,v^{\prime} = v/t,\mu^{\prime} = \mu /t,$ $\epsilon_{0}^{\prime } = \epsilon_{0} /t$, we finally obtain:

19 $$ \left( {\frac{\tau }{{\tau_{0} }}} \right)^{ - 1} = \frac{{v^{{{\prime }2}} n_{imp} }}{2\pi } \times \mathop \int \limits_{0}^{\pi } dk_{x} \mathop \sum \limits_{i = 1,2} \left( {\left( {h^{\prime}_{1} \left( {k_{x} ,y_{i} } \right)} \right)^{ - 1} + \left( {h^{\prime}_{2} \left( {k_{x} ,y_{i} } \right)} \right)^{ - 1} } \right)\left( {z_{i}^{{\prime }} } \right)^{ - 1} , $$

where $y_{i}^{{\prime }}$ is given by

20 $$ \begin{aligned} y_{i}^{{\prime }} & = \frac{{\mu^{{\prime }} - 2cosk_{x} \pm 2\sqrt {{\mathbb{C}}} }}{{1 + \left( {\alpha_{R}^{{{\prime }2}} + \beta_{D}^{{{\prime }2}} + 2\alpha^{{\prime }}_{R} \beta^{{\prime }}_{D} sink_{x} } \right)}},\quad {\mathbb{C}} = \left( {\alpha^{{\prime }}_{R}{\,}^{2} + \beta^{{\prime }}_{D}{\,}^{2} } \right)\sin^{2} k_{x} + \alpha^{{\prime }}_{R}{\,}^{2} + \beta^{{\prime }}_{D}{\,}^{2} + 4\alpha^{{\prime }}_{R} \beta^{{\prime }}_{D} \\ z_{i}^{{\prime }} & = \left( {1 - y_{i}^{{{\prime }2}} /4} \right)^{1/2} ,h^{{\prime }}_{1,2} \left( {k_{x} ,y_{i} } \right) = 1/{\mathbb{Z}},\quad {\mathbb{Z}} = \frac{{y_{i}^{{\prime }} \left( {2\alpha^{{\prime }}_{R} \beta^{{\prime }}_{D} \sin k_{x} + \left( {\alpha^{{\prime }}_{R}{\,}^{2} + \beta^{{\prime }}_{D}{\,}^{2} } \right)z_{i}^{{\prime }} } \right)}}{{8z_{i}^{{\prime }} \left[ {\left( {\alpha^{{\prime }}_{R} {\text{sin}}k_{x} + \beta^{{\prime }}_{D} z_{i}^{{\prime }} } \right)^{2} + \left( {\alpha^{{\prime }}_{R} z_{i}^{{\prime }} + \beta^{{\prime }}_{D} {\text{sin}}k_{x} } \right)^{2} } \right]^{1/2} }} \\ \end{aligned} $$

## (a) Charge current

When SOIs are present, the charge current operators $J^{c} { }$ can be obtained by differentiating the charge polarization operator with respect to time: ${\varvec{P}}^{c} = e\mathop \sum \limits_{{j_{x} ,j_{y} }} {\varvec{R}}_{{j_{x} ,j_{y} }} c_{{j_{x} ,j_{y} }}^{\dag } Ic_{{j_{x} ,j_{y} }}$ where ${\varvec{R}}_{{j_{x} ,j_{y} }}$ denotes the position observable. Here we work in the Heisenberg picture to calculate the currents. The charge current along the x and y axis is given in terms of the transformed operators as:

21 $$ J_{x,y}^{c} = \frac{{\partial {\varvec{P}}^{c} }}{\partial t} = i\left[ {H_{0} ,e\mathop \sum \limits_{{{\text{j}}_{{\text{x}}} ,{\text{j}}_{{\text{y}}} }} \vec{R}_{{{\text{j}}_{{\text{x}}} ,{\text{j}}_{{\text{y}}} }} {\text{c}}_{{{\text{j}}_{{\text{x}}} ,{\text{j}}_{{\text{y}}} }}^{\dag } I{\text{c}}_{{{\text{j}}_{{\text{x}}} ,{\text{j}}_{{\text{y}}} }} } \right] $$

which can be written as

22 $$ J_{x,y}^{c} = e/\hbar \mathop \sum \limits_{k} \left( {f_{1}^{x,y} \left( {\varvec{k}} \right)\alpha_{1,k}^{\dag } \alpha_{1,k} + f_{2}^{x,y} \left( {\varvec{k}} \right)\alpha_{2,k}^{\dag } \alpha_{2,k} + f_{3}^{x,y} \left( {\varvec{k}} \right)\alpha_{1,k}^{\dag } \alpha_{2,k} + f_{3}^{*x,y} \left( {\varvec{k}} \right)\alpha_{1,k}^{\dag } \alpha_{2,k} } \right), $$

where

22a $$ f_{1,2}^{x} \left( {\varvec{k}} \right) = 2tsink_{x} \mp \cos k_{x} \left( {i\alpha_{R} \left( {p_{{k_{x} ,k_{y} }}^{*} - p_{{k_{x} ,k_{y} }} } \right) + h_{D} \left( {p_{{k_{x} ,k_{y} }}^{*} + p_{{k_{x} ,k_{y} }} } \right)} \right), $$

22b $$ f_{1,2}^{y} \left( {\varvec{k}} \right) = 2tsink_{y} \mp \cos k_{x} \left( {i\alpha_{R} \left( {p_{{k_{x} ,k_{y} }}^{*} - p_{{k_{x} ,k_{y} }} } \right) + h_{D} \left( {p_{{k_{x} ,k_{y} }}^{*} + p_{{k_{x} ,k_{y} }} } \right)} \right), $$

22c $$ f_{3}^{x} \left( {\varvec{k}} \right) = - \cos k_{x} \left( {i\alpha_{R} \left( {1 + p_{{k_{x} ,k_{y} }}^{2} } \right) + \beta_{D} \left( {1 - p_{{k_{x} ,k_{y} }}^{2} } \right)} \right), $$

22d $$ f_{3}^{y} \left( {\varvec{k}} \right) = - \cos k_{y} \left( {i\alpha_{R} \left( {1 + p_{{k_{x} ,k_{y} }}^{2} } \right) + \beta_{D} \left( {1 - p_{{k_{x} ,k_{y} }}^{2} } \right)} \right), $$

### (b) Spin current

Similarly, to calculate the spin current density, we define spin polarization operator ${\varvec{P}}^{{s_{z} }}$ = $\mathop \sum \limits_{{j_{x} ,j_{y} }} {\varvec{R}}_{{j_{x} ,j_{y} }} s_{z} c_{{j_{x} ,j_{y} }}^{\dag } c_{{j_{x} ,j_{y} }}$, and the spin current is given by

23 $$ J_{x}^{{s_{z} }} = \frac{{d{\text{P}}^{{{\text{s}}_{{\text{z}}} }} }}{dt} = \frac{i}{\hbar } \left[ { {\text{P}}^{{{\text{s}}_{{\text{z}}} }} ,H_{0} } \right], = \mathop \sum \limits_{{j_{x} ,j_{y} }} {\varvec{R}}_{{j_{x} ,j_{y} }} \frac{{ds_{z} }}{dt} $$

where $\frac{{ds_{z} }}{dt} \equiv \hat{\user2{\tau }} = \frac{1}{i\hbar }[\hat{\user2{s}}_{{\varvec{z}}} , H_{0} ]$, ${\hat{\tau }}$ being related to the spin torque density ${\text{T}}_{{\text{z}}}$ through the relation: ${\text{T}}_{{\text{z}}} = {\uppsi }^{\dag } \left( {\text{r}} \right){{\hat{\tau }\psi }}\left( {\text{r}} \right)$. So the spin current is zero if the spin torque density is zero. Thus spin current introduced here arises from the spin torque caused by the spin–orbit interaction. To calculated the spin current we have used relation (23). Therefore, the x-component of the spin current in terms of operators $\alpha_{{\user2{k }}} \;{\text{and}}\;\alpha_{k}^{\dag }$, can be written as:

24 $$ \begin{aligned} J_{x}^{{s_{z} }} & = - 2t\mathop \sum \limits_{{k_{x} ,k_{y} }} \sin k_{x} \left( {\alpha_{R} \sin k_{y} + \beta_{D} \sin k_{x} } \right) \frac{1}{{\left| {\zeta \left( {\varvec{k}} \right)} \right|}} \alpha_{k}^{\dag } \sigma_{x} \alpha_{k} \\ & \quad + 2t\mathop \sum \limits_{{k_{x} ,k_{y} }} \sin k_{x} \left( {\alpha_{R} \sin k_{x} + \beta_{D} \sin k_{y} } \right)\left| {\zeta \left( {\varvec{k}} \right)} \right|^{ - 1} \alpha_{k}^{\dag } \sigma_{y} \alpha_{k} \\ & \quad + 2\mathop \sum \limits_{{k_{x} ,k_{y} }} \left( { \beta_{D}^{2} - \alpha_{R}^{2} } \right)\cos k_{x} sin^{2} k_{y} \left| {\zeta \left( {\varvec{k}} \right)} \right|^{ - 2} \\ & \quad \times \alpha_{k}^{\dag } \left[ {\begin{array}{*{20}c} {\left( {\beta_{D} - i\alpha_{R} } \right)p_{{k_{x} ,k_{y} }}^{*} } & {\left( {\beta_{D} + i\alpha_{R} } \right)p_{{k_{x} ,k_{y} }}^{2} } \\ { - \left( {\beta_{D} - i\alpha_{R} } \right)p_{{k_{x} ,k_{y} }}^{*2} } & {\left( {\beta_{D} + i\alpha_{R} } \right)p_{{k_{x} ,k_{y} }} } \\ \end{array} } \right]\alpha_{k} . \\ \end{aligned} $$

## Spin hall and charge conductivity

Next, to study the effect of Rashba and Dresselhaus SOIs and the static random impurities on the currents, we employ the Green function formalism of Kubo^38,39^. In this technique, the longitudinal charge conductivity is obtained from the imaginary part of the charge current–current correlation function

25 $$ \mathop \prod \limits_{c} \left( {i\omega_{n} } \right) = \frac{\hbar }{N}\mathop \int \limits_{0}^{\beta } d\tau e^{{i\omega_{n} \tau }} \left\langle {T_{\tau } J^{cx} \left( \tau \right)J^{cx} \left( {\tau^{{\prime }} } \right)} \right\rangle . $$

Conductivity is calculated using imaginary part of ${\Pi }\left( {i\omega } \right)$.

26 $$ \sigma_{xx} { } = { }\mathop {\lim }\limits_{\omega \to 0} Im\left[ {\frac{{{\Pi }_{c} \left( {i\omega_{n} } \right)}}{\omega }} \right]. $$

where $\omega_{n}$ represent the Matsubara frequency, $T_{\tau }$ is the imaginary time ordering, ${ } \beta = 1/\kappa_{B} T{ }$ is the Boltzmann factor, the brackets … represent the thermodynamic average and N is the number of lattice sites. The charge current at two different times can be obtained from Eq. (21) as

27 $$ \begin{aligned} \vec{J}_{x}^{c} \left( \tau \right) & = \mathop \sum \limits_{k} \left( {f_{1}^{x} \left( k \right)\alpha_{1,k}^{\dag } \left( \tau \right)\alpha_{1,k} \left( \tau \right) + f_{2}^{x} \left( k \right)\alpha_{2,k}^{\dag } \left( \tau \right)\alpha_{2,k} \left( \tau \right)} \right. \\ & \quad \left. { + f_{3}^{x} \left( k \right)\alpha_{1,k}^{\dag } \left( \tau \right)\alpha_{2,k} \left( \tau \right) + f_{3}^{*x} \left( k \right)\alpha_{1,k}^{\dag } \left( \tau \right)\alpha_{2,k} \left( \tau \right)} \right) \\ \end{aligned} $$

28 $$ \begin{aligned} \vec{J}_{x}^{c} \left( {\tau^{{\prime }} } \right) & = \mathop \sum \limits_{k} \left( {f_{1}^{x} \left( k \right)\alpha_{1,k}^{\dag } \left( {\tau^{{\prime }} } \right)\alpha_{1,k} \left( {\tau^{{\prime }} } \right) + f_{2}^{x} \left( k \right)\alpha_{2,k}^{\dag } \left( {\tau^{{\prime }} } \right)\alpha_{2,k} \left( {\tau^{{\prime }} } \right)} \right. \\ & \quad \left. { + f_{3}^{x} \left( k \right)\alpha_{1,k}^{\dag } \left( {\tau^{{\prime }} } \right)\alpha_{2,k} \left( {\tau^{{\prime }} } \right) + f_{3}^{*x} \left( k \right)\alpha_{1,k}^{\dag } \left( {\tau^{{\prime }} } \right)\alpha_{2,k} \left( {\tau^{{\prime }} } \right)} \right) \\ \end{aligned} $$

The expression for the current–current correlation function is calculated by substituting Eqs. (27) and (28) in Eq. (25):

29 $$ \begin{aligned} \mathop \prod \limits_{c} \left( {i\omega_{n} } \right) & = \frac{\hbar }{N}\mathop \int \limits_{0}^{\beta } d\tau e^{i\omega \tau } \left\langle {\left[ {\mathop \sum \limits_{{\text{k}}} f_{1}^{x} \left( k \right)^{2} \alpha_{{1,{\varvec{k}}}}^{\dag } \left( \tau \right)\alpha_{{1,{\varvec{k}}}} \left( \tau \right)\alpha_{{1,{\varvec{k}}}}^{\dag } \left( {\tau^{{\prime }} } \right)\alpha_{{1,{\varvec{k}}}} \left( {\tau^{{\prime }} } \right)} \right.} \right. \\ & \quad + \mathop \sum \limits_{{\text{k}}} f_{2}^{x} \left( k \right)^{2} \alpha_{{2,{\varvec{k}}}}^{\dag } \left( \tau \right)\alpha_{{2,{\varvec{k}}}} \left( \tau \right)\alpha_{{2,{\varvec{k}}}}^{\dag } \left( {\tau^{{\prime }} } \right)\alpha_{{2,{\varvec{k}}}} \left( {\tau^{{\prime }} } \right) \\ & \quad + \mathop \sum \limits_{{\text{k}}} f_{3}^{x} \left( k \right)f_{3}^{x*} \left( k \right)\alpha_{{1,{\varvec{k}}}}^{\dag } \left( \tau \right)\alpha_{{2,{\varvec{k}}}} \left( \tau \right)\alpha_{{2,{\varvec{k}}}}^{\dag } \left( {\tau^{{\prime }} } \right)\alpha_{{1,{\varvec{k}}}} \left( {\tau^{{\prime }} } \right) \\ & \quad \left. {\left. { + \mathop \sum \limits_{{\text{k}}} f_{3}^{x} \left( k \right)f_{3}^{x*} \left( k \right)\alpha_{{2,{\varvec{k}}}}^{\dag } \left( \tau \right)\alpha_{{1,{\varvec{k}}}} \left( \tau \right)\alpha_{{1,{\varvec{k}}}}^{\dag } \left( {\tau^{{\prime }} } \right)\alpha_{{2,{\varvec{k}}}} \left( {\tau^{{\prime }} } \right)} \right]} \right\rangle \\ \end{aligned} $$

**Figure 2:**
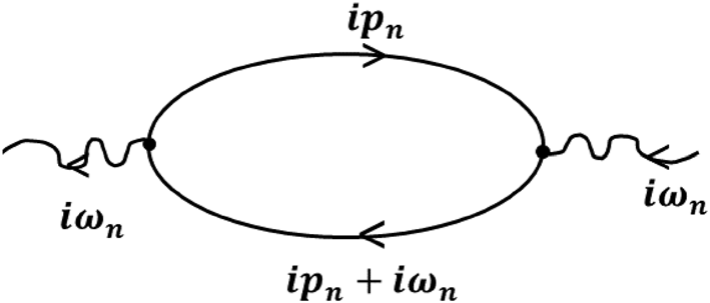
Bubble diagram for the current–current correlation function.

Equation (29) is solved in parts. We first calculate the correlation function corresponding to the first term of Eq. (29). Using Wick's theorem, the current–current correlation function for the first term in the Fourier basis is given by:

30 $$ \mathop \prod \limits_{c}^{{\prime }} \left( {i\omega_{n} } \right) = \frac{\hbar }{N}\frac{1}{\beta }\mathop \sum \limits_{{k,ip_{n} }} f_{1}^{x} \left( k \right)^{2} \mathop \int \limits_{0}^{\beta } d\tau G\left( {k,ip_{n} } \right)G\left( {k,ip_{n} + i\omega_{n} } \right), $$

where ${\text{ G}}\left( {{\text{k}},{\text{ip}}_{{\text{n}}} } \right)$ and ${\text{G}}\left( {{\text{k}},{\text{ip}}_{{\text{n}}} + {{{\text{i}}\omega }}_{{\text{n}}} } \right)$ are the single particle Green functions which are represented by the lower and upper lines of the loop of the bubble diagram for the current–current correlation function shown in Fig. 2. It can be seen that at the vertices where the electrons lines meet, the momentum is conserved. Here the two electron lines differ by the Matsubara frequency $i\omega_{n}$. In order to calculate the charge conductivity (CC) we calculate the imaginary part of the current–current correlation function and perform analytic continuation $\left( {{{{\text{i}}\omega }}_{{\text{n}}} \to {\upomega } + {{{\text{i}}\delta }}} \right)$ and write the Green functions in terms of spectral functions. Finally, we divide the correlation function by $\omega$ and in the limit ${\upomega } \to 0,$ CC for the first part of current–current correlation function is given by

31 $$ \sigma_{xx}^{c} = - \frac{\hbar }{N}\mathop \sum \limits_{k} f_{1}^{x} \left( k \right)^{2} \mathop \int \limits_{ - \infty }^{\infty } d\epsilon A\left( {k,\epsilon } \right)^{2} \left\{ {\frac{{\partial n_{F} \left( \epsilon \right)}}{\partial \epsilon }} \right\}. $$

Similarly, we can evaluate correlation functions for the other terms of Eq. (29) and the total CC assumes the following expression:

32 $$ \sigma_{xx}^{c} = - \frac{\hbar }{N}\mathop \sum \limits_{k} \int d\epsilon \left\{ {\frac{{\partial n_{F} \left( \epsilon \right)}}{\partial \epsilon }} \right\}\left[ {f_{1}^{x2} \left( k \right) A^{1} \left( {k,\epsilon } \right)^{2} + f_{2}^{x2} \left( k \right) A^{2} \left( {k,\epsilon } \right)^{2} + 2f_{3}^{x2} A^{1} \left( {k,\epsilon } \right)A^{2} \left( {k,\epsilon } \right)} \right], $$

where $A^{1} \left( {k,\epsilon } \right)\;{\text{and}}\;A^{2} \left( {k,\epsilon } \right)$ represent the spectral functions for the electrons. In the case of low impurity concentration, the spectral function is given by:

33 $$ \left( {A^{1,2} \left( {k,\epsilon } \right)} \right)^{2} = \left( {4\pi /\hbar } \right)\tau \left( {{\varvec{k}},\mu } \right)\delta \left( {\mu - \epsilon_{{{\varvec{k}}1,2}} } \right). $$

Using Eq. (33) in Eq. (32), the expression for longitudinal CC reduces to

34 $$ \sigma_{xx}^{c} = \frac{{4\pi e^{2} \tau }}{{\hbar \tau_{0} kT^{{\prime }} }}\mathop {\iint }\limits_{ - \pi }^{\pi } dk_{x} dk_{y} \mathop \sum \limits_{i = 1,2} \left( {\left( {f^{{\prime }}_{i,x} \left( {k_{x} ,y_{i} } \right)} \right)^{2} \times \exp \left( {\left( {\epsilon^{{\prime }}_{{i,{\varvec{k}}}} - \mu^{{\prime }} } \right)/kT^{{\prime }} } \right)/kT^{{\prime }} \left( {1 + \exp \left( {\left( {\epsilon^{{\prime }}_{{i,{\varvec{k}}}} - \mu^{{\prime }} } \right)/kT^{{\prime }} } \right)} \right)^{2} } \right), $$

Similarly, from the Kubo formalism, the expression for the spin Hall conductivity (SHC) is given by

35 $$ \sigma_{xy}^{{s_{z} }} = - \frac{1}{\pi }\sum \frac{{Im\left[ {\left\langle {\left| {J_{x}^{{s_{z} }} } \right|} \right\rangle \left\langle {\left| {J_{y}^{c} } \right|} \right\rangle } \right]}}{{\left( {\epsilon_{1,k} - \epsilon_{2,k} } \right)^{2} + \left( {\frac{1}{\tau }} \right)^{2} }}\left( {f_{E} \left( {\epsilon_{1,k} - \mu^{{\prime }} } \right) - f_{E} \left( {\epsilon_{2,k} - \mu^{{\prime }} } \right)} \right), $$

where $J_{x}^{{s_{z} }}$ is the x-component of the spin current and $J_{y}^{c}$ is the y-component of charge current. Using the value of $J_{y}^{c}$ and $J_{x}^{{s_{z} }}$ from Eqs. (22) and (23), the final expression for SHC is given by

36 $$ \sigma_{xy}^{{s_{z} }} = \frac{{\left( {\alpha^{\prime }_{R}{\,}^{2} - \beta^{\prime }_{D}{\,}^{2} } \right)}}{\pi } \times \mathop {\iint }\limits_{0}^{\pi } dk_{x} dk_{y} \frac{{\left( {f_{E} \left( {\epsilon_{1,k} - \mu^{\prime } } \right) - f_{E} \left( {\epsilon_{2,k} - \mu^{\prime } } \right) } \right)\cos k_{y} \sin^{2} k_{x} }}{{\left( { \left( {\left| {\zeta^{\prime } \left( k \right)} \right|} \right)^{2} + \left( {\tau_{0} /\tau } \right)^{2} } \right)\left( {\left| { \zeta^{\prime } \left( k \right)} \right|} \right)}}, $$

where $f_{E} \left( {\epsilon_{1,2,k} - \mu^{{\prime }} } \right)$ is the Fermi distribution and at zero temp it is given by $f_{E} (\epsilon_{1,2,k} - \mu^{{\prime }} ) = {\text{heaviside}}\;(\epsilon_{{\varvec{k}}} \pm 2\left| {\zeta ({\varvec{k}})} \right| - \mu^{{\prime }} ).$

## Numerical results discussion

**Figure 3:**
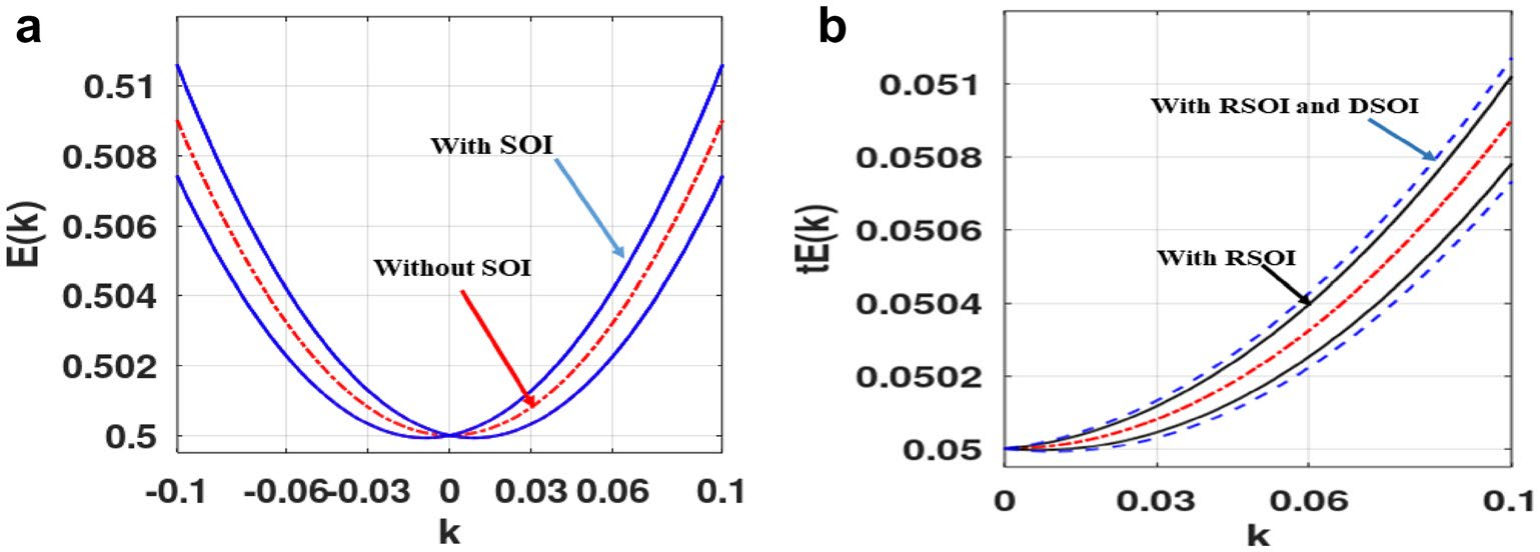
(**a**) Energy dispersion along the x-direction in k space. (**b**) For different values of $\alpha_{R} \;{\text{and}}\;\beta_{D}$. (Plotted using octave − 6.3.0).

We measure all energies in units of $$ t$$. Numerical computations have been done using the software GNU Octave version 6.3.0^40^. Figure 3 shows the energy dispersion along the $$k_{x}$$-direction in the presence and absence of SOI. The splitting of bands and the renormalization of the ground state energy (GS) are clearly visible.

**Figure 4:**
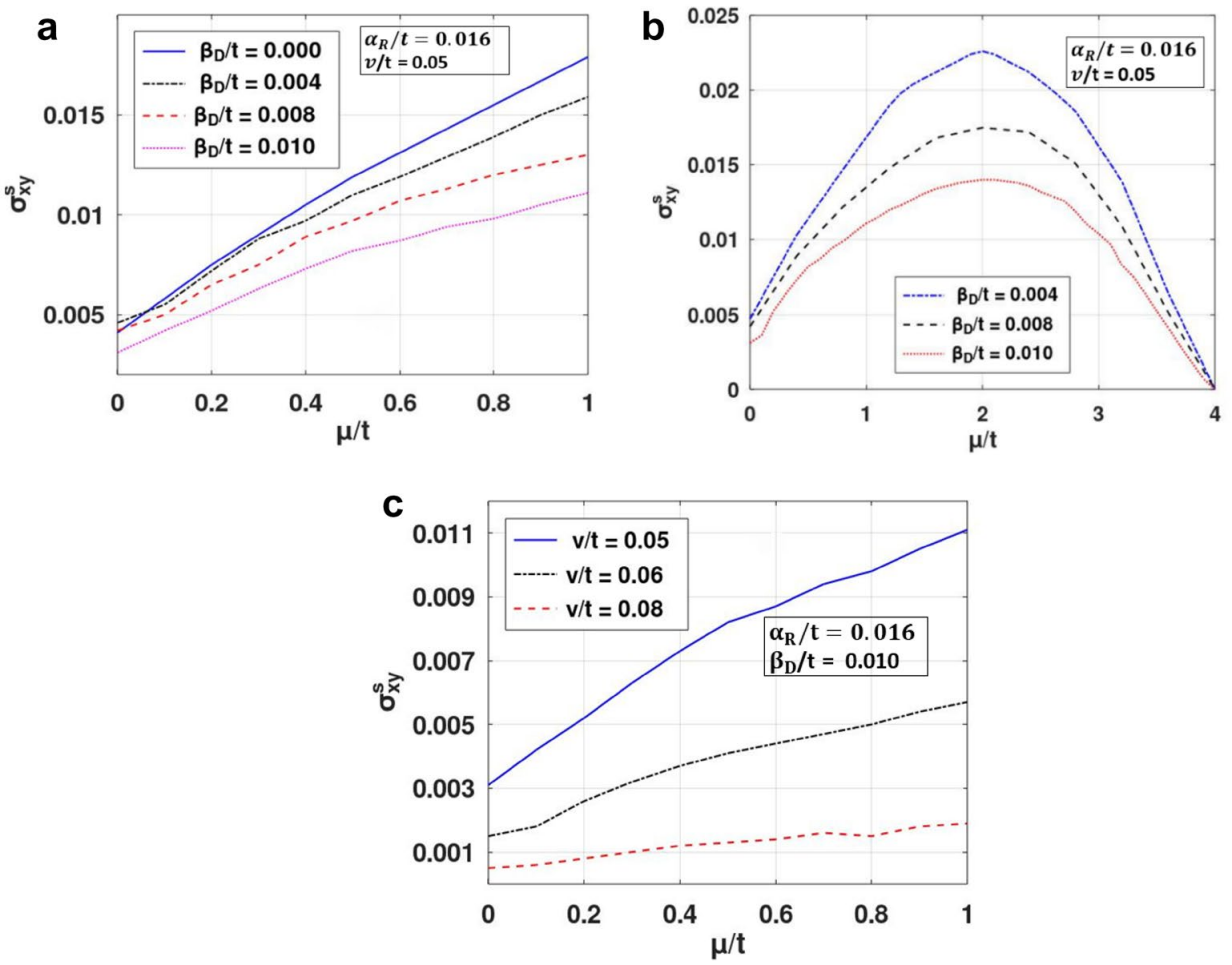
SHC versus $\mu /t$ for different values of: (**a**, **b**) $\beta^{{\prime }}_{D}$, (**c**) $v^{{\prime }}$. (Plotted using octave − 6.3.0).

**Figure 5:**
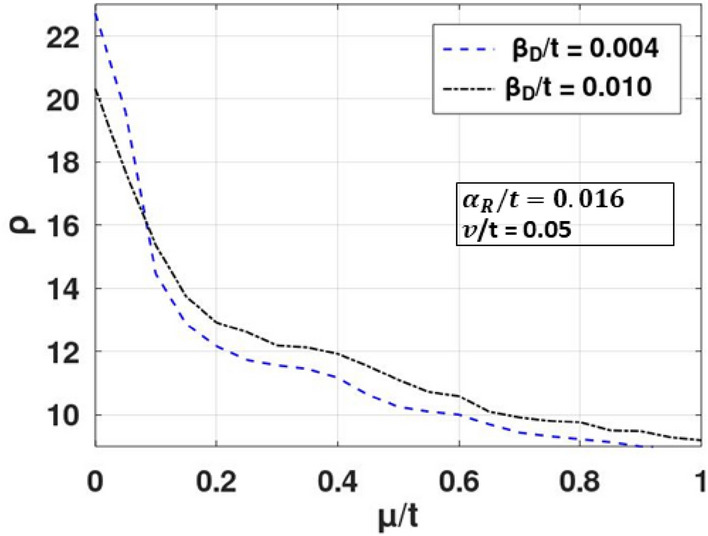
Density of state $\left( \rho \right)\;{\text{vs}}\;\mu /t$. (Plotted using octave − 6.3.0).

The effect of this splitting and the renormalization can be seen in Fig. 4, where SHC is plotted with respect to chemical potential. Initially SHC increases with $\mu$ (Fig. 4a), reaches a maximum at some crucial *μ-*value and then decreases with a further increase in *μ* to zero (Fig. 4b). When studied in the presence of the DSOI (Fig. 4a), SHC increases with *μ* at a lower rate as it counters the Rashba effect. This increase in SHC with chemical potential can be easily understood from Fig. 5. At low *μ*, the density of states is high which gives rise to more scattering events leading to a lower value in SHC. As *μ* increases, DOS decreases and we observe an increase in SHC. However, as μ increases beyond a certain value, DOS becomes very small (Fig. 5), and then not any states are available for conduction and SHC decreases with μ at large value of μ (Fig. 4b). As a result, SHC exhibits a maximum with respect to μ (Fig. 4b). Figure 4c shows the nature of the variation of SHC with respect to *μ* for different values of the impurity strength (v). As expected, with an increase in impurity strength, SHC decreases.

**Figure 6:**
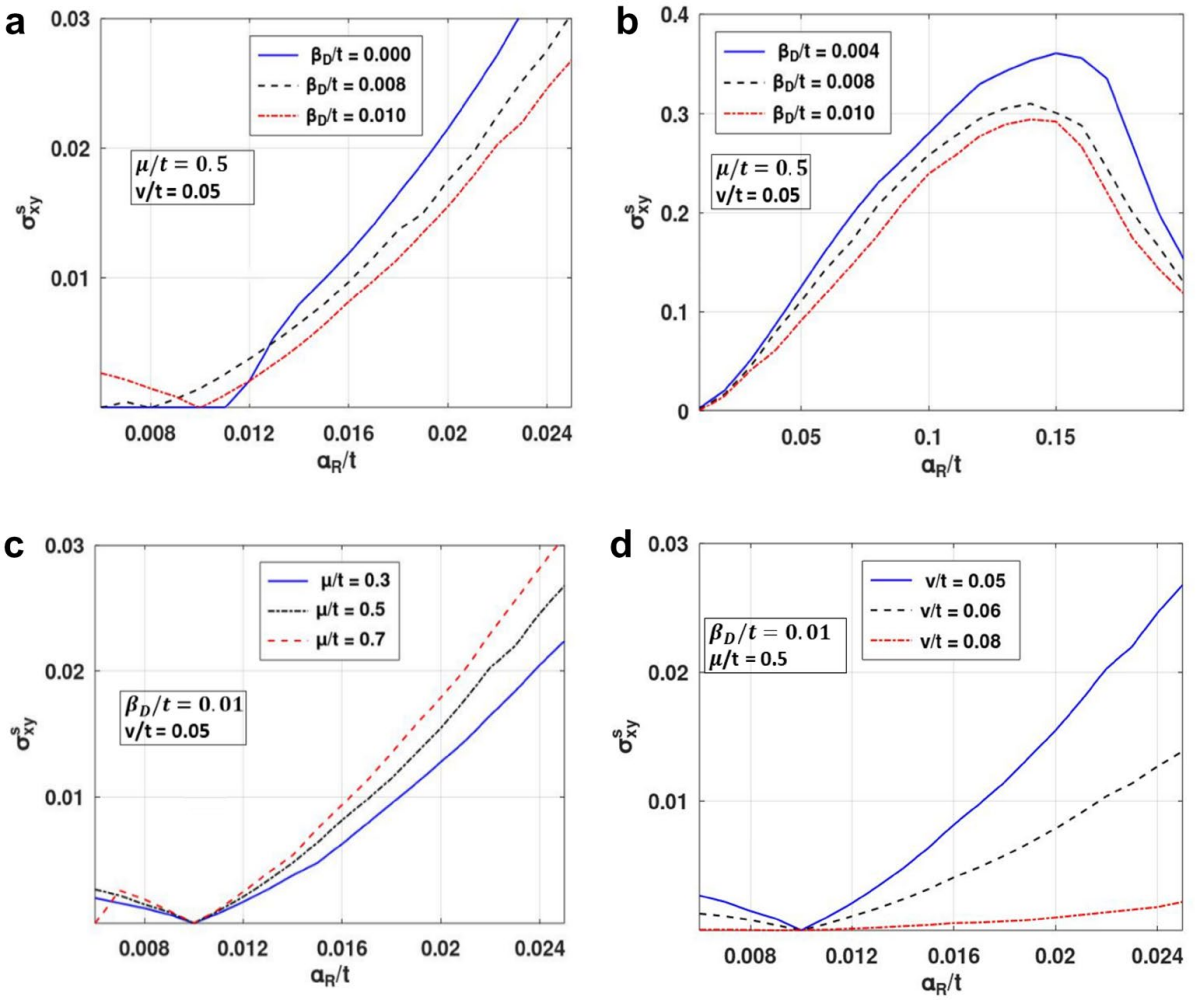
SHC versus $\alpha_{R} /t$ for different value of: (**a**, **b**) $\beta_{D} /t$, (**c**) $\mu /t$ and (**d**) ${\text{v}}/t$. (Plotted using octave − 6.3.0).

**Figure 7:**
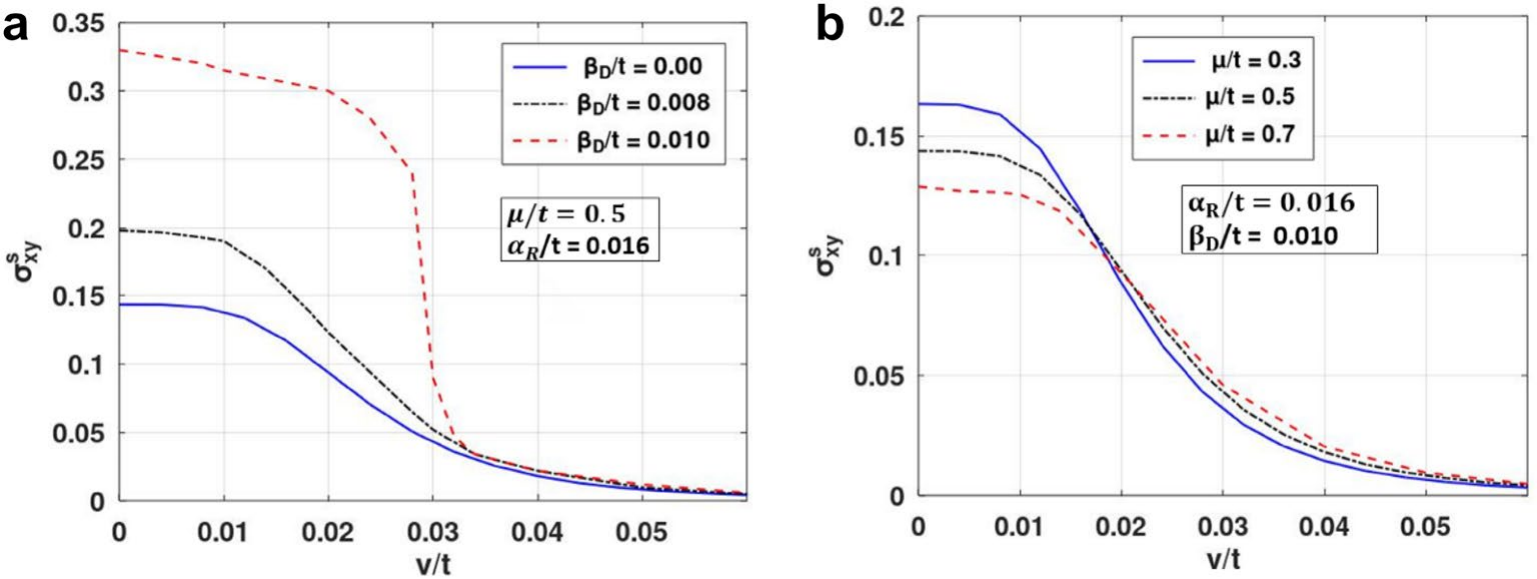
SHC versus ${\text{v}}/t$ for distinct: (**a**) $\beta_{D} /t$,and (**b**) $\mu$*/t* values. (Plotted using octave − 6.4.0).

In Fig. 6, we present the behaviour of SHC as a function of $\alpha_{R}$ with different values of SMT parameters. Figure 6a describes the behavior of SHC versus RSOI strength $\alpha_{R}$. One can see that at $\beta_{D} = 0$, SHC is zero up to a certain value of $\alpha_{R}$. For $\beta_{D} \ne 0,$ SHC has a finite value finite at $\alpha_{R} = 0$. This shows that the difference in the spin states is minimum at smaller value of $\alpha_{R}$ for $\beta_{D} = 0,$ while for finite $\beta_{D}$, there is an appreciable difference in the spin states that causes a larger spin current. As $\alpha_{R}$ increases, RSOI counters the effect of DSOI and concomitantly, SHC decreases. SHC becomes zero when $\alpha_{R}$ becomes equal to $\beta_{D}$. As $\alpha_{R}$ increases further, SHC increases with $\alpha_{R}$ (Fig. 6a). With the increase in $\alpha_{R}$ beyond $\alpha_{R} = \beta_{D}$, the spin up and down bands move apart, causing a higher spin imbalance and hence a rise in SHC. As $\alpha_{R}$ is increased further, SHC exhibits an asymmetric peak at a critical $\alpha_{R}$, and then decreases with further increase in $\alpha_{R}$ and finally reaches a constant saturation value (Fig. 6b). This peak mentioned above gives the maximum value of SHC for the system. As $\beta_{D}$ is increased, the spin imbalance decreases leading to a decrease in spin current. Figure 6c displays the behaviour of SHC as a function of $\alpha_{R}$ for a few values of $\mu$. The plot exhibits a similar nature as in Fig. 6a. SHC increases with the increase in $\mu$, which is also evident from Fig. 4. In Fig. 6d we examine the behaviour of SHC versus $\alpha_{R}$ for different values of the impurity strength. The figure shows that as the impurity strength increases, SHC decreases. In Fig. 7, SHC is considered as a function of impurity strength for various system parameters. When the strength of impurity scattering coefficient is very small, SHC remains essentially unaffected by impurity. As the impurity coupling increases, one can observe a gradual decrease in SHC. Figure 7a,b shows that with an increase in DSOI strength and chemical potential, SHC has a lower value and higher decreasing rate.

**Figure 8:**
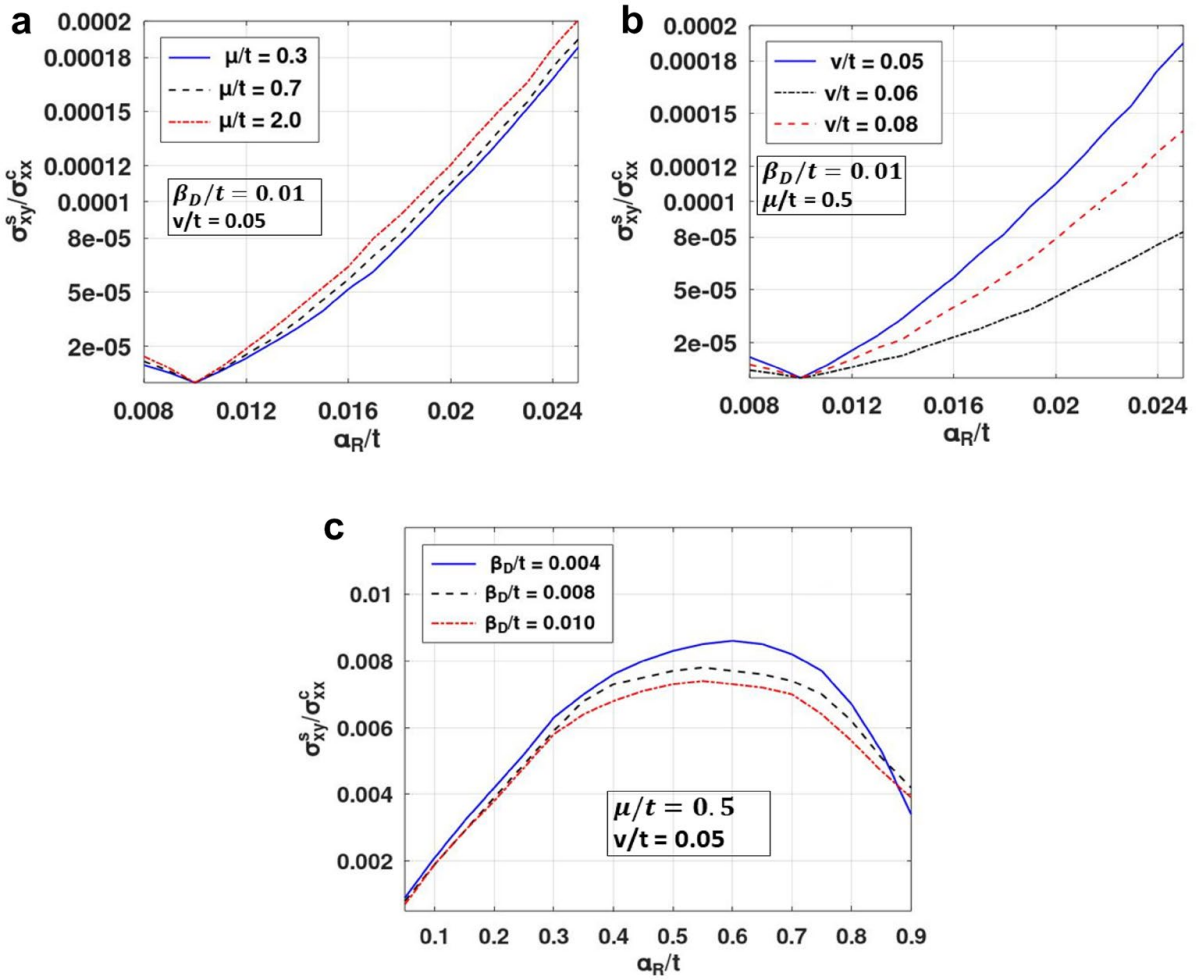
SHA versus RSOI coefficient for different value of*:* (**a**) $\mu /t$, (**b**) ${\text{v}}/t$ and (**c**) $\beta_{D} /t$. (Plotted using octave − 6.3.0).

Finally In the last segment, we present our results for the spin-Hall angle (SHA) which is measured by the ratio: SHC/LCC. The effect of RSOI, DSOI and the impurity on LCC was thoroughly investigated by us in our earlier works^34,35^. Figure 8a,b shows the plot of SHA versus $\alpha_{R}$ for different values of the chemical potential and impurity strength. SHC increase with increase in chemical potential while decrease with increase in impurity strength similar to SHC. And for higher value of Rashba strength once again one can observe a peak-like structure, which is maximum for a lower value of $\beta_{D}$ (Fig. 8c).

**Figure 9:**
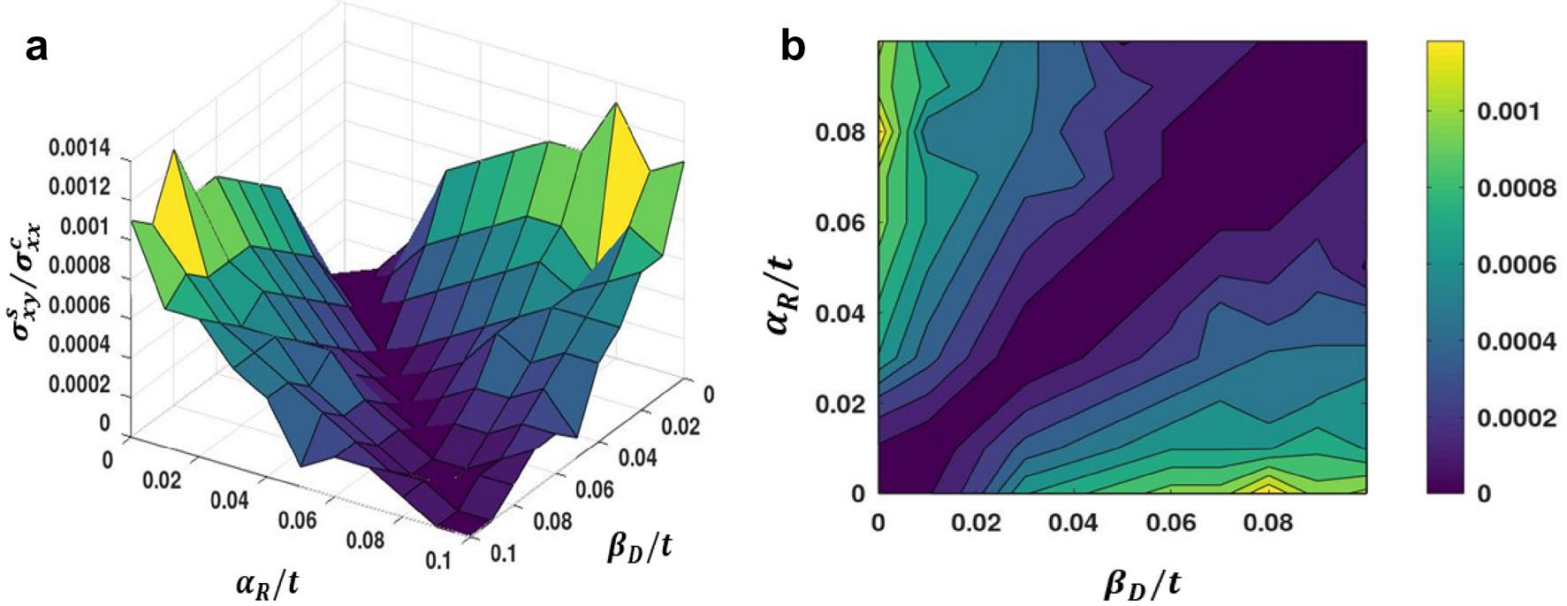
(**a**) SHC/LCC as a function of $\alpha_{R} /t\;{\text{and}}\;\beta_{D} /t$. (**b**) Contour plot of SHC/LCC in the $\left( {\alpha_{R} - \beta_{D} } \right) -$ plane for *μ/t* = 0.05 and *v/t* = 0.05. (Plotted using octave − 6.3.0).

**Figure 10:**
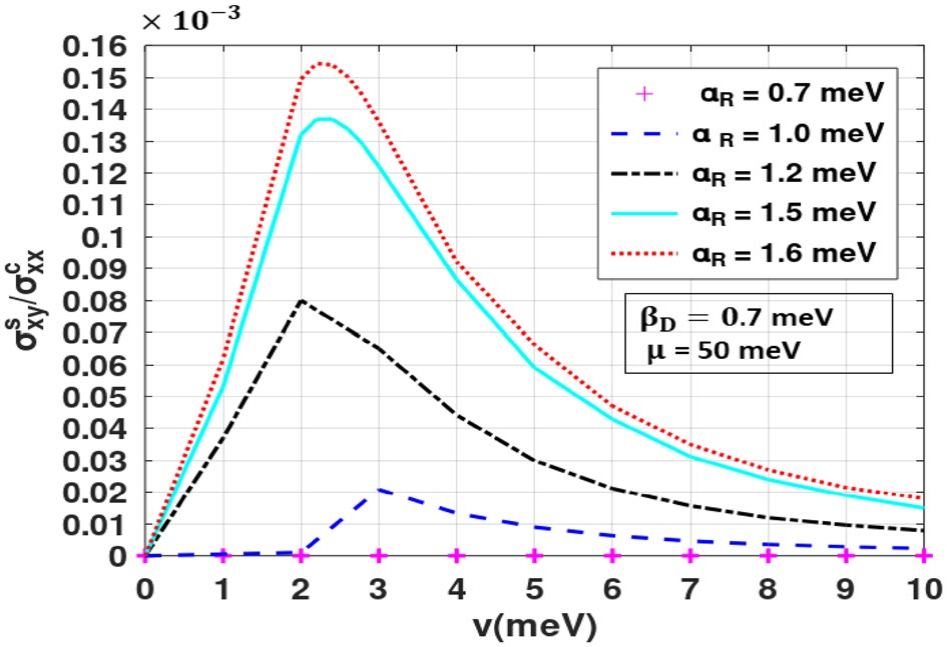
SHC/LCC as a function of $v$. (Plotted using octave − 6.3.0).

In Fig. 9, we show the three-dimensional and contour plots for the ratio of the spin Hall to charge conductivities as a function of $\alpha_{R} \;{\text{and}}\;\beta_{D}$. As anticipated, when $\alpha_{R} \;{\text{and}}\;\beta_{D}$ are small, the ratio of SHC to LCC is almost zero. As we increase any one of the SOI strengths (keeping the other constant), the ratios initially increase but beyond a critical value of $\alpha_{R}$ or $\beta_{D}$ they decrase. This gives rise to peak structures in SHC/LCC. When $\alpha_{R} \;{\text{and}}\;\beta_{D}$ are both increased together, the ratio remains zero, and as one becomes more effective than the other, we witness a measurable value. The Rashba strength can be adjusted up to 50% by varying the gate voltage^41–43^. Figure 10 shows the SHC/LCC ratio for Indium arsenide for which $\alpha_{{\text{R}}} \;{\text{and}}\;\beta_{{\text{D}}}$ can take values in the range: 0. 07–1.6 meV and $\mu$ can be in the range: 20–50 meV^39,44^. It can be seen that when impurity strength is low, SHA increases. This is because at low impurity concentration, SHC remains constant (Fig. 7) while LCC decreases^35^. From Fig. 10, one can see that for InAs, SHA can be in the range: 0.00001–0.00015.

## Conclusion

We have explored the nature of spin-torque-induced transport properties of a two-dimensional tight-binding system in the presence of Rashba and Dresselhaus spin–orbit interactions and random static impurities. In the absence of impurity scattering, the system admits an exact solution with two degenerate electron states. We have treated the electron-impurity scattering by the Matsubara Green function technique using the diagrammatic perturbation theory. The randomness of the impurities has been taken into account by performing configuration averaging. Finally, the Kubo formalism is used to calculate spin-Hall conductivity.

Our results suggest that as a function of the chemical potential, the spin-Hall conductivity increases monotonically for the smaller values has a peak structure for higher values. The peak values of these quantities, however decrease with the increase in the Dresselhaus coupling, as the DSOI broadens the bands. Similar nature is also observed with respect to the RSOI coefficient.

We have also studied the effect of the electron-impurity interaction strength v on SHC in the presence of both the RSOI and DSOI effects and observed that for low impurity strength, SHC remains almost negligible, but with an increase in the impurity strength, SHC decreases rapidly and the rate of decrease depends on the difference between $\alpha_{R}$ and $\beta_{D}$.

Finally, the spin-Hall angle has been calculated and it is shown that when one of the two spin–orbit interactions dominates, the spin-Hall angle increases and it vanishes whenever the Rashba and Dresselhaus interactions become equal. As an example, the variation of SHA as a function of the impurity coupling strength is shown for InAs.
